# Obstacles to preventing obesity in children aged 2 to 5 years: Latino mothers’ and fathers’ experiences and perceptions of their urban environments

**DOI:** 10.1186/s12966-017-0605-9

**Published:** 2017-11-02

**Authors:** Carlos Penilla, Jeanne M. Tschann, Emma V. Sanchez-Vaznaugh, Elena Flores, Emily J. Ozer

**Affiliations:** 1 School of Public Health, University of California, Berkeley, 50 University Hall, Berkeley, CA 94720-7360 USA; 2 Department of Psychiatry, University of California, San Francisco, Box 0848, San Francisco, CA 94143-0848 USA; 3 Health Education Department, San Francisco State University, 1600 Holloway Ave, San Francisco, CA 94132 USA; 4 Department of Counseling, University of San Francisco, 2130 Fulton St., San Francisco, CA 94118 USA

**Keywords:** Latino, Childhood obesity, Parent feeding behavior, Urban environments, Fathers

## Abstract

**Background:**

The prevalence of obesity among Latino children is alarmingly high, when compared to non-Latino White children. Low-income Latino parents living in urban areas, even if they are well-educated, face obstacles that shape familial health behaviors. This study used qualitative methods to explore parents’ experiences in providing meals and opportunities to play to their children aged 2 to 5 years. In contrast to most prior studies, this study examined perceptions of familial behaviors among both mothers and fathers.

**Methods:**

An ecological framework for exploring the associations of parental feeding behaviors and children’s weight informed this study. An interview guide was developed to explore parents’ experiences and perceptions about children’s eating and physical activity and administered to six focus groups in a community-based organization in the Mission District of San Francisco. Transcripts were coded and analyzed. Twenty seven mothers and 22 fathers of Latino children ages 2 to 5 participated.

**Results:**

Mothers, fathers, and couples reported that employment, day care, neighborhood environments and community relationships were experienced, and perceived as obstacles to promoting health behavior among their children, including drinking water instead of soda and participating in organized playtime with other preschool-age children.

**Conclusions:**

Results from this study suggest that the parents’ demographic, social and community characteristics influence what and how they feed their children, as well as how often and the types of opportunities they provide for physical activity, providing further evidence that an ecological framework is useful for guiding research with both mothers and fathers. Mothers and fathers identified numerous community and society-level constraints in their urban environments. The results point to the importance of standardized work hours, resources for day care providers, clean and safe streets and parks, strong community relationships, and reduced access to sugar-sweetened beverages in preventing the development of obesity in preschool-age Latino children.

## Background

Addressing childhood obesity is an urgent public health concern. In the United States, the prevalence of overweight and obesity among children is especially high in the Latino population [1]. In 2011–2012, 17% of Latino children ages 2 to 5 were considered obese, as defined by a body mass index (BMI) greater than or equal to the 95th percentile, compared to 4% of non-Latino White children of similar ages [1]. In the short term, obese children are at higher risk for health problems such as hypertension, type 2 diabetes, sleep disordered breathing and asthma, fatty liver disease, and abnormalities in menstruation and early menarche [2]. Obesity research and interventions with preschool-age Latino children have primarily focused on mother-child dyads [3]. While these interventions can be beneficial, an unintended negative consequence is the potential blaming of mothers for their children’s overweight without considering key factors beyond the dyad. Empirical evidence suggests that structural and environmental factors such as the cost of food, junk food advertising, the abundance of fast food, lack of places to exercise, and traffic or crime-related safety may be associated with obesity-related health disparities, especially among disadvantaged populations [4, 5]. No previous research has explored structural and environmental factors as related to parent-child relationships and how these factors may shape both mothers’ and fathers’ decisions and behaviors about what preschool-age Latino children eat or how they play.

### Framework

Using a socioecological approach to understanding familial health behaviors, we conceptualized children’s eating and physical activity routines as shaped by parents’ experiences and cultural traditions, as well as by environmental conditions outside of the parents’ control [6, 7]. These environmental conditions may support and or constrain parental goals of raising healthy children [8, 9]. A growing body of research has linked healthy behaviors with built, social and socioeconomic environmental assets (access to parks, social ties, affluence); similarly, unhealthy behaviors have been linked with built environmental obstacles (access to fast food outlets), suggesting that neighborhood environments are an important level at which to intervene to prevent childhood obesity [5, 10]. For example, a recent study of racial/ethnic and socioeconomic characteristics in urban environments suggests that Latino families may not have access to either supermarkets or grocery stores [11, 12]. These factors and other features, such as accessibility to high-density fast food, may create obesogenic environments that influence parents’ decisions and behaviors.

**Fig. 1:**
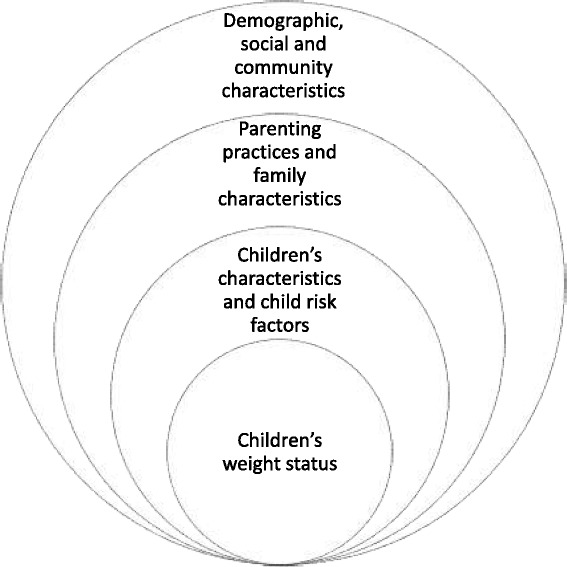
Graphic based on Davison and Birch’s (2001) ecological framework for exploring factors associated with children’s weight status

This qualitative study was informed by Davison and Birch’s (2001) ecological framework of childhood obesity, which poses that three contextual layers influence the development of overweight: 1) child’s characteristics and risk factors, 2) parenting practices and family characteristics, and 3) demographic, social and community characteristics (see Fig. 1). This study focused on the broader social and environmental influences, namely, how urban neighborhoods influence children’s weight status through parents’ child-feeding and physical activity routines.

### Why explore both mothers’ and fathers’ child-feeding behaviors?

While mothers can play a dominant role in child feeding, fathers play a role as well [13–15]. Nevertheless, fathers are seldom included in pediatric obesity research [16, 17]. The U.S. Department of Labor reported that mothers spend more time on childcare tasks than fathers do, but the time gap between full-time working mothers and full-time working fathers continues to narrow [18]. Because the majority (66%) of Latino children live in two-parent families [19], Latino fathers play a role in two-parent households. Moreover, nationally, the share of Latino single-mother households (22%) and Latino single-father households (24%) is similar, and part of a growing national trend in single-parent households [20].

Studies conducted with Latino mothers about their role in feeding have primarily focused on the needs of mother-child dyads within the home and school environments [21]. Results from these studies generally indicate that mothers describe their primary responsibility as feeding the family, but that in addition to the cost of food, mothers may experience various obstacles outside the home, such as unappealing lunches provided to children at school, proximity to grocery stores, junk food advertising, busy schedules, and in some low-income neighborhoods, unsafe streets and parks [22–26]. A qualitative study with Latino mothers reported that some children come home and request specific foods they perceive as healthy and/or refuse foods that parents offer if they perceive them as unhealthy, because of what they are taught in school about nutrition, as well as what they are offered for school lunch [23]. Unfortunately, some children may come home hungry from school because they did not get enough to eat at lunch or found the lunch choices unappealing. Results from a recent study exploring obstacles to low-income Latino and African American mothers’ preferred child feeding indicated that parents in neighborhoods without grocery stores take public transportation and/or carpool to other neighborhoods to buy healthful food, making it challenging to offer healthful food [24]. For immigrant mothers, the feeling of isolation that develops as a result of cultural differences between their country of origin and the U.S. and loss of social support were also reported as obstacles to feeding children healthful foods. When these factors interact, they may limit newly immigrated mothers’ opportunities to adapt to the local community and navigate through its resources. There is extensive research on obstacles to feeding children healthful food within the home and school environment, but there is a dearth of research about how community, demographic and social characteristics in underserved urban communities influence eating and drinking, especially among Latino children.

Although research with mothers has helped our understanding of some of the factors that influence parents’ behavior, studies have often not included fathers. In order to inform effective obesity prevention interventions among preschool-age Latino children, research is needed that moves beyond mother-child dyads at home and school. To our knowledge, no qualitative studies on broader environmental obstacles to preventing childhood obesity have reported on the experiences and perspectives of both mothers and fathers of preschool-age Latino children. This study addresses this gap by investigating Latino mothers’ and fathers’ experiences and their perceptions about the ways in which community and environmental factors influence the development of obesity among children aged 2 to 5 years. The overall research question was: What social and environmental characteristics in urban neighborhoods influence familial relationships and make it harder for children to eat healthful food and stay physically active, and how do these characteristics operate?

With the goal of informing future community-level research aimed at preventing obesity among preschool-age children living in low-income urban neighborhoods, this research used focus group methodology to examine mothers’ and fathers’ perceived environmental obstacles to preventing obesity and the influence of these obstacles on children’s eating and physical activity. A major aim of focus group methodology is to shed new light on a group of people’s understanding of a phenomenon, such as the eating behavior of children living in urban environments, by using group interaction to produce data and insights that might otherwise not emerge in individual interviews [27]. Focus group interviews with Latino parents living in urban areas allowed for in-depth group discussion of unique and shared experiences and perceptions. Another benefit of focus groups is that it helps prevent the power dynamics between interviewer and interviewee that can emerge in one-to-one interviews, and encourages a freer flow of communication among the members in the group. For example, having the parents talk amongst themselves about healthy eating allowed freer dynamics to emerge and gave space for parents to agree and disagree with each other; thus, deepening the discussion.

## Methods

### Participants

Using a purposive approach to sample selection [28, 29], the present study recruited 49 Latino parents (27 mothers and 22 fathers) to participate in six focus groups. Parents were recruited from throughout San Francisco, with emphasis on the Mission District, because it has the largest Latino population in the city (approximately 38% of local residents are Latino) [30], and has multiple organizations that serve low-income Latino families with preschool-age children. Most referrals to the focus groups were provided by personnel working at community-based health centers and immigrant resource centers, who distributed study flyers to their clients with children. Trained bilingual research assistants screened potential participants who called the research offices in response to recruitment efforts. Eligible participants were the parent/guardian of a child between ages 2 to 5, spoke English or Spanish and were of Mexican, Guatemalan or Salvadoran descent (see Table 1). These three major ethnic groups make up over 82% of the Latino population in the San Francisco area [31]. This research was approved by the university’s institutional review board.

**Table 1:** Characteristics of Mexican, Guatemalan and Salvadorian origin parents who participated in child obesity focus groups

	Mothers (*n* = 27)	Fathers (*n* = 22)
	% or M (SD)	% or M (SD)
Country of birth^a^		
USA	19%	23%
Mexico	37%	41%
El Salvador	19%	9%
Guatemala	15%	9%
Honduras	7%	5%
Nicaragua	4%	14%
Spanish is primary language	59%	46%
# of years lived in the US	16 (11.2)	19 (8.0)
Age (years)	30 (5.8)	35 (9.1)
Currently employed	33%	68%
Years of education	12 (3.7)	11 (2.3)
Relationship status		
Single/Divorced	34%	32%
Married/Living with sig. other	66%	68%

^a^Though a few participants were born in Honduras and Nicaragua, they reported that they were of Mexican, Guatemalan or Salvadorian descent

### Focus groups

The focus groups were conducted in the conference room of a community-based organization in San Francisco in 2011 and 2012. This organization was chosen for its central and accessible location in a neighborhood with a high concentration of Latino families [25]. A total of six focus groups were conducted, two with mothers-only (English, *n* = 7; Spanish, *n* = 12), two with fathers-only (English, *n* = 8; Spanish, *n* = 6), and two with couples (English, *n* = 8; Spanish, *n* = 8). We included both single and married mothers and fathers in order to obtain a broader view on preventing obesity. Focus group facilitators were matched by gender. Couples focus groups were co-led by a female and male facilitator. The first author participated as facilitator in the two fathers-only and two couples focus groups. Three focus groups were conducted in English and three were conducted in Spanish. The focus groups lasted approximately two hours and were audiotaped. Focus group participants were given $50 for their participation and $30 to assist with childcare while attending the focus groups.

A focus group guide was developed to help obtain information on promoting healthful food and healthy weight in urban areas. The initial questions and probes were used to explore parental concerns about childhood obesity and how parents determine the type and amount of food to offer children on a daily basis. For example, “In your neighborhood, how much do parents with Latino children ages 2 to 5 talk about concerns about childhood overweight or eating better?” and “How do parents figure out how much to feed their children as they grow older?” (see Table 2). The remaining three questions and probes were used to explore parents’ perceptions of factors that are highly correlated with child weight such as soda consumption, physical activity routines and sedentary behavior including questions about experiences related to maintaining healthful behavior [32]. For example, “If a family likes to drink soda, what would make it harder to drink less?” and “Is there anything that makes it easier for families to do physical activity with their children?” (see Table 2). The focus group guide was translated side-by-side for accuracy. The audiotapes of the groups were transcribed in their original language and checked for accuracy against original recordings.

**Table 2:** Focus group guide

1. Overweight: In your neighborhood, how much do parents with Latino children ages 2 to 5 talk about concerns about childhood overweight or eating better? What do they say?
2. Children’s meals: How do parents figure out how much to feed their children as the children grow older?
Probe: a) If some days family members, or another person feeds the child, how do parents figure out how much their child has eaten that day?
3. Soda: If a family likes to drink soda, what would make it harder for them to drink less soda?
Probes: a) What would make it easier for a family to drink less soda?
4. Physical activity: How much do parents with Latino children ages 2 to 5 talk about physical activity?
Probes: a) Is there anything that makes it easier for families to do physical activity with their children?; b) Is there anything that could be done in neighborhoods to make it easier for families to do physical activity?

### Qualitative analysis

Consistent with guidelines for qualitative analysis described by Miles and Huberman (1994), we used a multi-stage analytic process that combined deductive, inductive and verification techniques to strengthen the reliability of the coding system and validity of the findings. In the initial stage, the authors read the transcripts and summary notes to generate an initial set of descriptive phrases that would be used to code the transcript text. In order to establish consistent application of codes to text, two trained Spanish-bilingual research assistants coded the six transcripts. Using an iterative process to strengthen the reliability of the coding system [28, 29], the first two transcripts of two focus groups (one in English and one in Spanish) were coded in increments of about 10 pages at a time and immediately reviewed to examine closely the agreement between the two research assistants. Any inconsistencies in the coded text were discussed and resolved by the authors at each interval, to ensure clear inclusion and exclusion criteria. This process resulted in changes to the initial codes, such as expanding on initial two-word themes, and in changes to how the remaining four transcripts were coded by the two research assistants.

With respect to the deductive process, some codes were developed based on focus group questions reflecting the conceptual framework, such as environmental features including liquor stores and fast food availability. In terms of the inductive process, we developed codes to reflect new themes that emerged from the focus group discussions, such as emigrating to the U.S. and fears of deportation. In the next stage, transcripts with coded text were uploaded into QSR NVivo in order to generate groupings of coded passages and to facilitate further analysis and interpretation. Software was used to connect relevant data segments with each other, forming new categories, clusters and networks of information for analysis in order to draw new conclusions [28, 29, 33]. The coded transcript data from the six focus groups were then sorted by codes and analyzed by the authors to identify the larger emergent themes and to organize the results. In the verification stage, all transcripts and resultant data analyses were reviewed by the authors to confirm accuracy of reported conceptual relationships between the themes.

## Results

Most participants were born outside the U.S. (81% of mothers and 77% of fathers; see Table 1). Generally, mothers had lived in the U.S. for 16 years (SD = 11.2) and had completed 12 years of education (SD = 3.7). Fathers had lived in the U.S. for an average of 19 years (SD = 8.0) and had completed an average of 11 years of education (SD = 2.3). Most mothers were unemployed (67%), while most fathers were employed (68%).

**Fig. 2:**
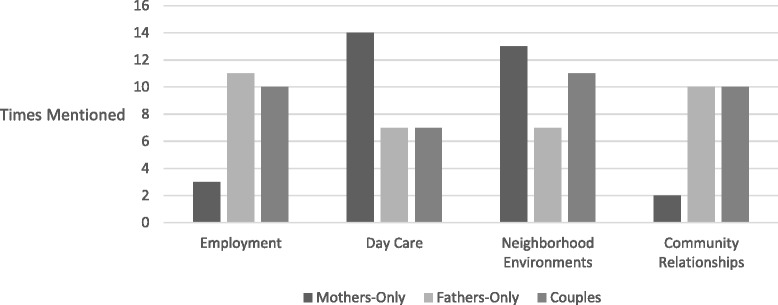
Frequency of four structural themes (employment, day care, neighborhood environments and community relationships) by mothers-only, fathers-only and couples focus groups

Results are presented following the ecological framework of childhood obesity [9], from individual to community levels, but organized according to broader social and environmental characteristics. This study reports on four structural categories relevant to the ecological framework of childhood obesity which guides this study: employment, day care, neighborhood environments and community relationships (see Table 3). Except for employment, which was not mentioned by mothers in the English-language mothers-only group, all themes were mentioned in all groups so results are presented together. The relative emphasis of themes by group type is described at the end of the results section (see Fig. 2). The results from the Spanish and English-language focus groups were similar and are presented together (all quotes have been translated into English).

**Table 3:** Eleven themes and three subthemes from focus groups

Employment
1. Work is a parent’s highest priority.
2. Exhausting work schedules make it hard to prevent childhood overweight.
3. A bad economy, insufficient income and food price sensitivity influence parents’ ability to promote their preferred home and food environment.
Day care
1. Preschool policy influences what children ate at home.
2. Preschools can do more to help parents keep their children at a healthy weight.
a. Food provided to children in family-based day care is often unhealthy.
3. Schools should provide more opportunities for children to participate in physical activity.
Neighborhood environments
1. Liquor stores, fast food and soda are everywhere and influence what children eat.
a. Soda is addictive.
2. Advertisements influence what children want to consume and ultimately what they eat.
3. Dirty, unsafe neighborhood streets and parks are obstacles to children’s health.
a. Relatives help by providing children opportunities for physical activity.
Community relationships
1. Neighborhood structural factors influence community relationships.
2. Compared to their country of origin, parents experience a lack of social support from neighbors in the U.S., which limits children’s access to healthful food and physical activity.

### Structural obstacle 1: Employment

Discussions in this category included work as the highest priority, exhausting work schedules, a bad economy, and sensitivity to food and drink prices. Parents reported that spending time with their children was key to getting them to eat healthful food and to participate in physical activity. However, a bad economy and insufficient income were obstacles.

### Work is a parent’s highest priority

When asked about concerns about childhood overweight, parents reported that their highest priority is to provide for the family: for example, “Where I work there is a big Latin community, and it’s a lot of single mothers, or single fathers who come in and they have children that are very obese, and their thing is, ‘We work all day.’” Parents reported that work is a higher priority than their health. One father stated, “One thing I’ve learned is that the working man….won’t take time off to spend with his family unless he’s hurt, or sick… You see them working, he’ll get two jobs and work.”

### Exhausting work schedules make it hard to prevent childhood overweight

Parents reported that exhausting work schedules reduced parenting time, which in turn put their children at risk for obesity. One mother stated, “In the United States…people go to work, they have their own problems, the rent, the bills, the telephone, as a result, sometimes we don’t have time for our children, what we do is place them in day care or with the baby sitter.” Parents reported that work schedules disrupted child-feeding routines, and that having two jobs limited playtime with children. One mother stated, “But also because of the lifestyle that I have had, I am a salesperson…I have to leave for work, all of this creates a nutritional imbalance.” One father stated, “It’s all the Latinos, they got two jobs, so as long the kid is fed they’re fine, but if their kid is not doing anything athletic then the little kid is going to get chubby, he is just going to be bored watching TV, being on the computer and eating.”

### A bad economy, insufficient income and food price sensitivity influence parents’ ability to promote their preferred home and food environment

When discussing childhood overweight, parents reported that the high cost of food can lead to the purchase of unhealthy food, especially since quantity matters to big families. One parent stated, “It’s hard to…move towards healthy living when their environment…you go to McDonalds, buy a meal, $4 compared to making, buying a healthy meal will cost $10, they [parents] know it’s healthier for their kids, but…how is it going to hurt me financially?” Parents also indicated that a bad economy meant parents were experiencing increased financial and time constraints on their parenting practices. One father stated, “We are in a bad economy, you are either trying to get your kid to day care, rushing to find a job, or going to work. It’s kind of rough right now, I don’t think anybody…is talking about [childhood overweight].”

### Structural obstacle 2: Day care

Discussion in this category included preschool policy and family-based day care. Parents reported that they used multiple types of day care and that providers often differed from parents, as well as other providers, in terms of child-feeding behaviors, including the types of foods and drinks given to children. Someone other than a parent routinely helped feed young children.

### Preschool policy influences what children ate at home

When asked about children’s meals, parents reported that preschool health and food policy had both good and bad influences on what and how much their children ate at home. One mother stated, “Well, my son goes to a preschool that’s in my neighborhood and they have a… healthy snack policy… So for me sometimes it is really difficult because sometimes when I make their lunch with a fruit snack, which are like gummy bears, they…don’t allow that.” Conversely, parents reported that preschool feeding schedules included up to five meals per day, in addition to food eaten at home. One mother stated, “My child is two years old, weighs 40 pounds and eats five times per day because of the routine at day care, they [children] eat breakfast, a snack, lunch, a snack, an afternoon meal, and they eat a little bit of everything, when they are at home.” Parents explained that sometimes children came home hungry, because they did not like the school food. One parent stated, “Our daughter also goes to preschool and we always ask her when we pick her up, we pick her up after lunchtime, at noon, so I ask ‘What did you eat?’, and she says ‘I did not eat because I did not like the food.’”

### Preschools can do more to help parents keep their children at a healthy weight

Parents commented that preschools offered processed high fat foods to children and wanted preschools to provide all healthy food options. One father stated, “Food in schools and high schools, to be honest, has been bad, with high fat content and carbohydrates, very bad.” Parents said that preschool staff should give parents more information: for example, “I believe that parents are unaware of what they [children] ate…they [preschool personnel] tell you either that your child ate or that they ate well, but…parents don’t know what they ate if they are not with them.”

### Food provided to children in family-based day care is often unhealthy

Parents were aware and worried that grandmothers and/or eldest children, while valued as child care providers, were often an unhealthy influence on what children ate. One mother stated, “When I’m not at my house he asks, ‘Where’s mommy?’ and my parents are like, ‘She’s at work’ and he’s like, ‘Yay, I get to eat ice cream for breakfast.’” One father stated, “My grandma watches my kid sometimes…she gives them whatever they ask for, which is not good, but, it’s like other people that are not your parents, they tend to show a little more leniency on them, ‘Oh you want ice cream? Okay, go ahead.’”

### Schools should provide more opportunities for children to participate in physical activity

When discussing day care, parents added that they wanted schools to provide more opportunities for preschool-age children to participate in physical activity. One father stated, “A lot of teachers will just throw them the ball and say, ‘go have fun at recess.’ You got to be creative and make it fun, so everybody participates and nobody feels like they are better than the other.” Another parent stated, “It’s really hard for the Hispanic community since sometimes parents work 16-18 hours a day and don’t have time after they get home, tired, but one thing that we can do as a community is to push our schools, so they can have physical education.”

### Structural obstacle 3: Neighborhood environments

Discussion in this category included the abundance of liquor stores, fast food and soda, and dirty, unsafe parks. Parents reported that society, and neighborhood stores and restaurants in particular, influences what their children consume. They also said that soda was unhealthy, addictive and everywhere.

### Liquor stores, fast food and soda are everywhere and influence what children eat

When asked about soda, parents reported that not all neighborhoods were the same and that their neighborhoods had an over-abundance of liquor stores, fast food and soda. One parent stated, “Every corner store is a liquor store, or fast food, and it’s hard when your kids leave the house, you don’t know what they are going to be exposed to, when a lot of kids come back home, they want a can of coke, cause all day that’s what we see in the streets.” Parents reported soda was common at parties. One mother stated, “I try to tell the kids no soda, no soda, but then we go to a party and there’s nothing but soda and there’s nothing else to drink. We’ll drink water. So it’s hard.”

### Soda is addictive

Parents reported that soda was unhealthy and addictive and it was hard to stop drinking it. One father stated, “I’m going to stop drinking soda, but then my family member is constantly drinking soda, it’s just like an addict, you try to quit a drug, but your best friend that you’re with all the time is constantly doing it, so you can’t give up that addiction.” To emphasize this point, one parent suggested putting limits on the amount of soda that can be purchased and consumed at one time, “This is going to sound crazy, but there are limits for wines, there are limits for beer, there are limits for almost everything, but except for soda. Like in my country, in Mexico they drink 10 beers and that’s all you can have…it’s almost the same like here, DUI or whatever.”

### Advertisements influence what children want to consume and ultimately what they eat

Parents reported that there are more advertisements for processed food and sugar-sweetened beverages than for free options, such as tap water, which in turn impacted what children consumed at home. One mother stated, “Because advertisements are everywhere, stores and restaurants. They don’t say have a Big Mac and some water, they say have a Big Mac and a coke.” Parents reported that TV ads targeted children and that they had a strong influence on what their children wanted to consume. One father stated, “You see all these messages on TV, like McDonalds get a 32 ounce coke, but you don’t see any bad commercials about soda, what it does.” To emphasize this point, parents suggested that soda marketing to children should be stopped: for example, “Talk to the Coca-Cola factories and tell them to stop promoting it.”

### Dirty, unsafe neighborhood streets and parks are obstacles to children’s health

When asked about physical activity, parents reported that cities like San Francisco have many resources, but obstacles such as high participation fees prevented family participation. Parents also said that they wanted structural changes such as more clean and safe parks in order to prevent childhood obesity. Parents reported that under-policing and park conditions require additional vigilance if and while their children use a park: for example, “You see broken glass, you see a basketball, but no rims, the parks are empty…they [parents] just rather keep their kids at home because of gang violence, drugs…they buy them a TV, play station, Xbox, or computer, as long as they don’t bother nobody.” Another parent stated, “Sometimes when we go to the park, we are there for like two to three hours and we never see a policeman, like coming around and checking, you know, ‘How’s everything?’ never, we have been living there for almost two years.”

Parents added that small apartments and small yards limit their children’s ability to do sufficient physical activity, which in turn increased their stress level and reduced their desire to eat. One mother stated, “It is very small where I live…not much space for playing, I am constantly tripping on a toy car or a shoe, boy does it get me upset.”

### Relatives help by providing children opportunities for physical activity

Parents reported that relatives and friends increased opportunities for their children’s physical activity. One mother stated, “Well my five-year old, there’s in-laws downstairs, so they play a lot, because the other kids are his age, so they are always playing on their scooters and their go-carts. My oldest takes them out and they go walk the dog.”

### Structural obstacle 4: Community relationships

Discussion in this category included the importance of neighborhood structural factors for building community relationships and a lack of social support from neighbors. Parents reported that they wanted help to improve community relationships, which in turn would help improve the environment for their children to grow up healthily.

### Neighborhood structural factors influence community relationships

Parents shared that families fought over limited public spaces, which in turn hurt rapport with neighbors. One parent stated, “My brother used to work at the Boys and Girls Club…that place got so packed with different nationalities and there became like racial fights…they stopped taking their kids, so it’s real limited, you know, resources for kids to play.” Parents reported that neighbors had different physical activity preferences, which made it hard to build rapport. One mother stated, “People [in my neighborhood]…don’t really go outside and play, they go to a school or a park.”

Parents expressed that a bad economy influenced community relationships, because it placed much stress and time-constraints on their social lives. One father stated, “I mean there is no work, so people are out there looking for jobs, you don’t have time to be socializing about stuff like this [childhood overweight].”

### Compared to their country of origin, parents experience a lack of social support from neighbors in the U.S., which limits children’s access to healthful food and physical activity

Parents who immigrated to the U.S. reported that neighborhoods in their country of origin were often places where everyone knew each other and where they had a feeling of belonging to a community, which in turn strengthened their family. One father stated, “In our countries, children played together, the neighbors’ doors were always open and we didn’t have any problems… Here…I don’t feel safe letting my daughter play outside, because I don’t know the neighbors.” In contrast to their country of origin, immigrant parents reported that neighborhoods in the U.S. are not built for developing community relationships: for example, “We can’t let our kids go out, because they’re going to get kidnapped. In our country, our kids could run up and down…everybody knew each other…over here, you see somebody coming, they lock the door, shut their shades.” Immigrant parents reported that fears of deportation narrowed their family’s ability to open up to other parents and fully engage in the community with their children. One father stated, “A large part of the city, the people, Latinos, many of them are here, let’s say undocumented, they live here in a state of fear which prevents them from relating with others, they live in a world with fewer options, a world that is more closed off.”

### Differences between mothers-only, fathers-only and couples focus groups

Based on focus groups with mothers-only, fathers-only and couples, we found that all categories of themes were discussed in all three types of groups, but the frequency with which some categories were discussed varied somewhat (see Fig. 2). We were unable to separate out mothers and fathers in the couples focus groups. As illustrated in Fig. 2, overall, mothers in the mothers-only focus groups raised themes of day care and neighborhood environments more than fathers, and fathers in the fathers-only focus groups reported employment and community relationships more than mothers. Fathers and couples mentioned employment more than mothers. We note that mothers in the English-language mothers-only group did not discuss employment. Mothers had twice as much to say about day care compared to both fathers and couples. Mothers also talked about neighborhood environments more than fathers and couples, while fathers and couples had the most to say about community relationships.

## Discussion

Results from this study suggest that community, demographic and social characteristics influence what parents feed their children and opportunities for physical activity, providing evidence that an ecological framework is useful for guiding research with both mothers and fathers. Moreover, study results illuminate the processes within parent-child relationships that may be related to child feeding in the context of these broader environmental factors. In contrast to an individual-level interpretation, the study findings shed light on Latino parents’ child-feeding decisions in environments that parents identify as lacking options for fresh food, having an overabundance of processed food, and an absence of clean and safe recreational areas. For many Latino families in urban areas, such structural factors are likely to undermine healthful decisions and behaviors, by creating chronic barriers to feeding children healthful food and keeping them active. These results point to Latino parents’ awareness of environmental factors, which can help in the development of obesity interventions addressing these issues. The study also reinforces the need for an ecological perspective on obesity prevention, as well as the need for interventions that target multiple factors that influence children’s health, including parents’ work structure, the varying quality of day care, unsafe neighborhood environments, and poor community relationships. Latino populations tend to live in neighborhood environments with fewer supermarkets, recreational opportunities, and these families typically have fewer socioeconomic resources; thus, compared to non-Latino populations, Latino families are likely to be disproportionately affected by the barriers identified in this study. On average, our study sample had resided in the U.S. for more than 15 years; thus, we would expect the structural obstacles we identified to be worse for less educated and recent immigrant families. Future research should examine environmental factors that may affect more recent immigrants. Furthermore, additional research is needed to identify new strategies for overcoming structural risk factors for obesity and supporting parents’ child-feeding behaviors in urban environments.

Employment. Our findings suggest that working multiple jobs influence what and how parents feed their children as well as reduce the time available for modeling their behaviors. Similar to some studies conducted with mothers, work schedules are exhausting and place time constraints on both mothers’ and fathers’ ability to provide healthful meals on a daily basis for their children. Lower wages require that parents take on more than one job to meet financial obligations, which reduces their personal time and prevents them from spending more time with their children. These findings have important implications for research and interventions related to the role of parental employment in childhood obesity prevention overall, such as addressing the impact of work schedule instability and unpredictability, especially among populations that work two or more jobs.

Day care. Additionally, our study suggests that day care influences what and how children are fed and that these factors are typically out of the parents’ control. For example, parents employ day care, neighbors and relatives to provide child care, which involves providing meals during the day, but feeding behaviors differ among providers. Furthermore, children’s meals away from home are not always healthy. These results imply that over time, preschool-age children who are fed by various providers may develop poor eating habits that may lead to the development of obesity by the time they enter school. Because many children are routinely fed outside of the home and typically not by a parent, future interventions in day care settings should consider partnering with parents to determine menus and eating schedules. Future research could explore methods for helping busy parents monitor their children’s meals for the entire day, every day during this critical developmental time.

Neighborhood environments. Liquor stores, fast food and soda were easily accessible and influenced parents’ decisions and behavior regarding what children ate. Additionally, dirty, unsafe parks in their neighborhoods restricted parents’ ability to offer opportunities for play and were barriers to children’s overall health. These findings are consistent with existing research indicating that behavioral targets for childhood interventions should continue to include avoiding sugar-sweetened beverages, reducing exposure to food marketing by decreasing screen time, and replacing sugar, and fried and empty-calorie foods with fruit, and vegetables at all meals and all snacks [34]. In order to encourage families to be physically active in urban areas, cities, parks and police departments should coordinate resources to provide children with safe environments when not at home or at school, such as providing clean sand boxes and regular police patrolling at public recreation areas.

Community Relationships. Parents not born in the US highlighted that they have lost social support as a result of migration and struggle with building new social support networks in their current neighborhoods. This loss of social support networks, plus fears of deportation among some family members leads parents to feel isolated and alone, and may have a negative effect on their parenting practices, including feeding practices. Similar to studies conducted with mothers who immigrated to the U.S. [24], these factors are perceived by immigrant parents in our study as limiting opportunities to develop relationships with other families; thus, making it challenging to fully adapt to the local culture and to successfully navigate through the local resources, including stores and organized sports programs. Local events that encourage both mothers and fathers to meet might influence family decisions about allowing children to go outside and play with the neighbors’ kids. Future research could explore how events such as school, park and community fairs could help create safe places for mothers and fathers to enhance social support focused on obesity prevention.

This is the first qualitative study conducted with both mothers and fathers of preschool-age Latino children to investigate their experiences and perceptions about feeding children healthy food, providing opportunities for physical activity and keeping them at a healthy weight. By including both mothers and fathers, this research contributes to the literature by illuminating parents’ beliefs and behaviors that could be targets of interventions to reduce the health risks associated with obesity, and improve the health status of Latino children regarding food, healthy feeding and opportunities to play. The themes from the mothers-only, fathers-only, and couples focus groups had some overlap and also some unique contributions. The frequency with which each type of focus group mentioned each theme suggests that all parents face similar obstacles, but that their influence is perceived to be more or less critical, depending on a parent’s role in the family. When examining the focus group themes by parents’ gender, it appears that mothers and fathers each have a somewhat distinct perspective and role in helping children stay at a healthy weight. While mothers mainly perceived structural characteristics, such as day care and neighborhood environment as obstacles to preventing obesity, fathers perceived employment and community relationships as the main obstacles. Future research could examine more closely why and how unpredictable work shifts and community relationships influence the child-feeding behaviors of fathers, especially single fathers and couples in order to understand their role, and to potentially identify new pathways for improving the socio-cultural and physical environment in urban areas to change the risk status of Latino children.

Strengths of this study include a methodological approach well-suited to providing a contextualized understanding of both Latino mothers’ and fathers’ experiences with raising preschool-age children in urban environments, and rigorous attention to increasing the trustworthiness of our findings by taking systematic steps to establish the reliability of the analysis among coders and the validity of our interpretation of data [29]. Focus groups have some potential disadvantages, such as the possibility that some parents may not have felt comfortable sharing their experiences in a group setting, or that some participants can take on more dominating roles and influence the expression of others in the group. It should be noted that we did not detect such occurrences in our reading of the transcripts. Consent to conduct follow-up interviews was not obtained so we were not able to assess whether the identified themes resonated with focus group participants. Qualitative data helps to capture some, but not all contextual information that helps explain the influences of neighborhood and familial resources on the meals and physical activity provided to Latino children ages 2 to 5 [35]. Other qualitative methods such as participant observation could help further explain how broader environmental factors influence eating and playing. Using different techniques such as surveys or individual interviews and controlling for neighborhoods with different socioeconomic characteristics could offer new, complementary results that could be useful in identifying factors that may influence obesity in preschool-age Latino children [36]. Our qualitative approach to discovering parents’ experiences and perspectives about children’s eating and physical activity offers important information that can be used to design future research and inform obesity prevention interventions in this population.

## Conclusions

Results from this study suggest that both mothers and fathers perceive that what Latino preschool-age children are fed, as well as their physical activity opportunities are influenced by their community, demographic and social characteristics. Parents often know what to do, but the results of this study point to the importance of critical structural factors such as standardized work hours and increased resources for day care providers to supporting their cultural practices to promote child health overall. Moreover, urban communities need clean, safe streets and parks, plus strong community relationships so that parents can feed children healthful food and provide physical activity opportunities. While most research on childhood obesity has focused on the child or mother-child dyads, this study found that mothers, fathers, and couples identified numerous community and society-level constraints on their ability to promote their preferred health behaviors and prevent obesity in children aged 2 to 5 years.
